# RF induced heating of pacemaker/ICD lead-tips during MRI Scans at 1.5T and 3T: evaluation in cadavers

**DOI:** 10.1186/1532-429X-18-S1-O121

**Published:** 2016-01-27

**Authors:** Volkan Acikel, Patrick Magrath, Scott E Parker, Peng Hu, Holden H Wu, J Paul Finn, Daniel B Ennis

**Affiliations:** 1 grid.19006.3e0000000096326718Department of Bioengineering, University of California Los Angeles, Los Angeles, CA USA; 2 grid.19006.3e0000000096326718Department of Radiological Sciences, University of California Los Angeles, Los Angeles, CA USA

## Background

Studies about the safety of MRI exams for patients with pacemakers/ICDs at 1.5T have been reported [Nazarian S., et al. Annals of internal medicine 155.7 (2011): 415-424.]. Of most concern is possible heating of the lead-tips in contact with the myocardium. Little is known about the relative safety of 3T MRI exams for these patients and in vivo lead-tip heating data is difficult, if not impossible, to obtain. Our objective was to measure lead-tip heating directly in human cadavers with pacemakers/ICDs at both 3.0T and 1.5T.

## Methods

Cadavers (N = 5, 3 male, Table 1, part 1) with existing pacemakers had fiberoptic temperature probes implanted adjacent to right atrial (RA), right ventricular (RV) and/or abandoned lead-tips under x-ray guidance. Whole-body CT was used to estimate lead-tip to probe-tip distances. Cadavers were exposed to 15-minutes of 4 W/kg whole body SAR at both 1.5T and 3T (Siemens Avanto and Prisma) for five isocenter positions: 6 cm superior to the chin (LM_1_) and in four 15 cm increments inferior to LM_1_ (LM_2_ to LM_5_) in order to evaluate lead-tip heating as a function of the device's position relative to isocenter (i.e. different MRI exams). Maximum temperature increases at the lead-tip were reported as △T_Max_ (temperature difference between the baseline before the MRI sequence and peak heating after 15-minutes).

**Table 1:** Part 1 is the table that shows vital statistics of the cadavers. Part 2 is the table that shows the lead tip to probe tip distances

Part 1	Gender	Weight (kg)	Height (cm)	BMI	Age			
Cadaver 1	Male	54	167	19.4	93			
Cadaver 2	Female	36	147	16.7	99			
Cadaver 3	Male	58	177	18.5	85			
Cadaver 4	Male	45	172	15.2	93			
Cadaver 5	Female	68	167	24.4	87			
Part 2	Cadaver 1	Cadaver 2 RA lead	Cadaver 3	Cadaver 4 RA lead	Cadaver 4 RV lead	Cadaver 5 RA lead	Cadaver 5 RV lead	Cadaver 5 Abandoned lead
Lead tip to probe tip distance	6.4 mm	3.4 mm	10 mm	2 mm	9 mm	6.5 mm	7.6 mm	4 mm

## Results

**Figure 1:**
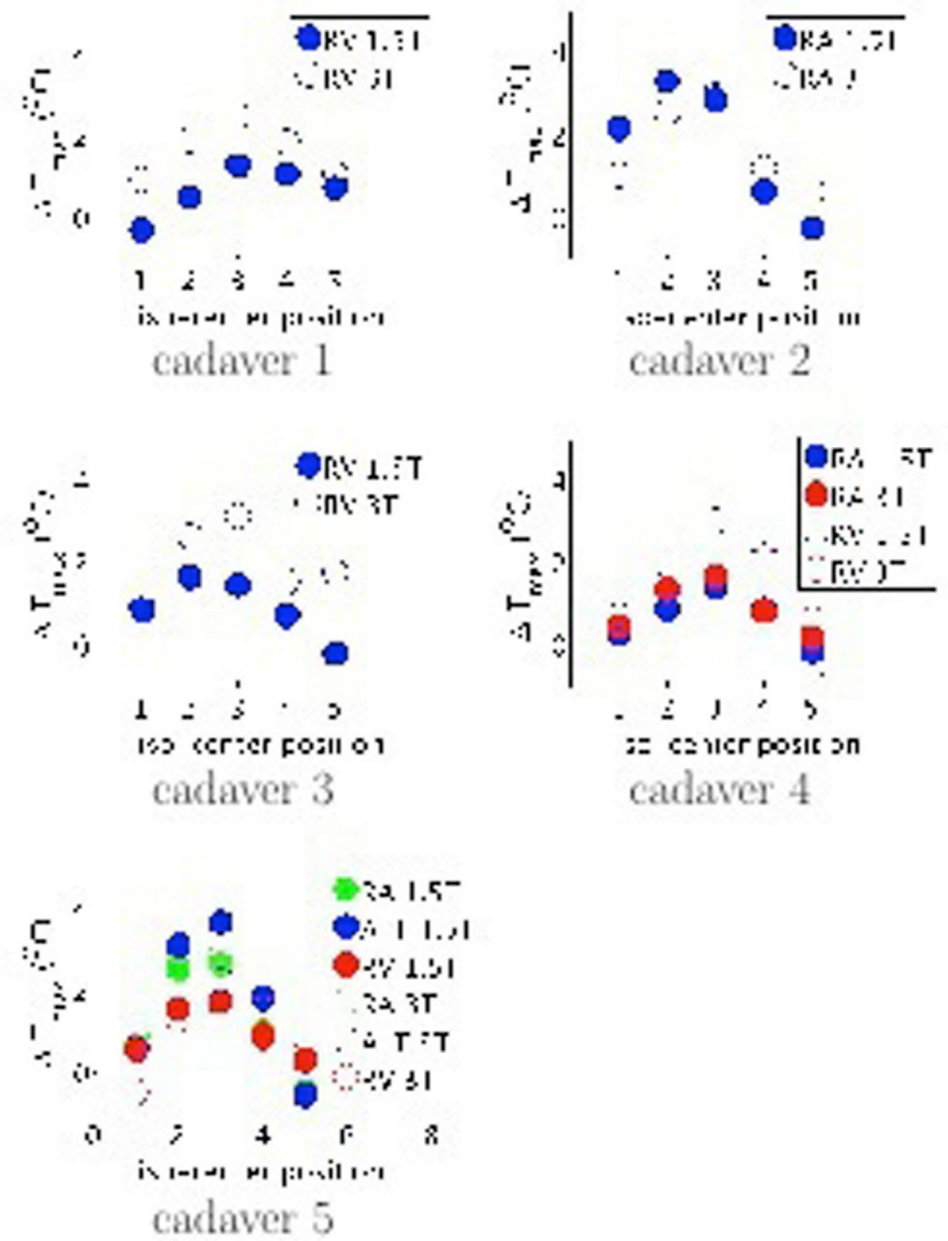
**△T**_**Max**_ **for each isocenter position**. Cadavers 1 and 3 have single chamber pacemakers (RV leads); cadavers 2, 4, and 5 have dual chamber pacemakers (RA and RV lead). Cadaver 5 also had 10 abandoned leads. For single chamber pacemakers a temperature probe was placed at the lead-tip and the remaining temperature probes were placed in remote tissue for reference. For dual chamber pacemakers, the temperature probes were placed at the RA and RV lead-tips and two probes were placed in remote tissue for reference. For the cadaver with abandoned leads two temperature probes were placed close to the lead-tips connected to the pacemaker and one probe was placed close to lead-tip of one abandoned lead (ALT). Note that in cadaver 2 the RV temperature probe was not close enough to the RV lead-tip (accidentally partially withdrawn) to obtain temperature data.

All temperature probes were ≤10 mm from the lead-tip (Table 1, part 2). Figure 1 shows △T_Max_ for 1.5T and 3T for each probe. Maximum heating was observed at the LM_2_ and LM_3_ isocenter positions for which the whole implant was inside the body transmit coil. △T_Max_ was >2C for 7 of 25 cases at 1.5T and for 12 of 25 cases at 3T, but never exceeded 4C.

## Conclusions

These data do not indicate a substantial difference between lead-tip heating at 1.5T and 3T, nor do they indicate △T_Max_>4C despite the lack of cooling due to tissue perfusion. Continued evaluation is warranted and on-going.

